# Single-cell transcriptome reveals diversity of Müller cells with different metabolic-mitochondrial signatures in normal and degenerated macula

**DOI:** 10.3389/fnins.2022.1079498

**Published:** 2022-12-22

**Authors:** Bei Liu, Jiali He, Ling Zhong, Lulin Huang, Bo Gong, Jing Hu, Hao Qian, Zhenglin Yang

**Affiliations:** ^1^Sichuan Provincial Key Laboratory for Human Disease Gene Study, Sichuan Provincial People’s Hospital, University of Electronic Science and Technology of China, Chengdu, China; ^2^School of Medicine, University of Electronic Science and Technology of China, Chengdu, China; ^3^Research Unit for Blindness Prevention of Chinese Academy of Medical Sciences (2019RU026), Sichuan Academy of Medical Sciences, Chengdu, China

**Keywords:** Müller cell, mitochondrial function, age-related macular degeneration, gliosis, macula

## Abstract

Müller cell is the most abundant glial cell in mammalian retina, supporting the functions of photoreceptors and other retinal neurons *via* maintaining environmental homeostasis. In response to injury and/or neuronal degeneration, Müller cells undergo morphological and functional alternations, known as reactive gliosis documented in multiple retinal diseases, including age-related macular degeneration (AMD), retinitis pigmentosa, diabetic retinopathy, and traumatic retinal detachment. But the functional consequences of Müller glia cell reactivation or even the regulatory networks of the retinal gliosis are still controversial. In this study, we reveal different subpopulations of Müller cells with distinct metabolic-mitochondrial signatures by integrating single cell transcriptomic data from Early AMD patients and healthy donors. Our results show that a portion of Müller cells exhibits low mitochondrial DNA (mtDNA) expressions, reduced protein synthesis, impaired homeostatic regulation, decreased proliferative ability but enhanced proangiogenic function. Interestingly, the major alternation of Müller cells in Early AMD retina is the change of subpopulation abundance, rather than generation of new subcluster. Transcription factor enrichment analysis further highlights the key regulators of metabolic-mitochondrial states of Müller glias in Early AMD patients especially. Our study demonstrates new characteristics of retinal gliosis associated with Early AMD and suggests the possibility to prevent degeneration by intervening mitochondrial functions of Müller cells.

## Introduction

Mitochondria is the major resource of ATP production in human tissues. Independent from cell nucleus, mitochondrion has its own genome encoding key subunits of oxidative phosphorylation system (OXPHOS) (Mercer et al., 2011). Moreover, OXPHOS also requires more than 80 proteins encoded by nuclear DNA (nDNA) (Mercer et al., 2011; Medini et al., 2021). The normal activities of mitochondria mainly depend on the coordination between expressions of both mitochondrial DNA (mtDNA) and nDNA (Barshad et al., 2018; Medini et al., 2021). It has been demonstrated that the levels of mtDNA are always positively correlated with the energy production and consumption of host cells (Mercer et al., 2011; Zhang et al., 2019), indicating that the metabolic-mitochondrial signature could be an indicator of cellular functions and statuses.

Retina is one of the highest energy-demanding organs in human beings (Joyal et al., 2018). Not surprisingly, different metabolic system disorders, especially the dysfunction of mitochondria, are always tied with various retinal diseases (Ferrington et al., 2020; Pfeiffer et al., 2020). Age-related macular degeneration (AMD) is one of the leading causes of visual impairment among the elderly population (Telegina et al., 2018; Heesterbeek et al., 2020). Although numerous risk factors have been identified, the mechanisms inducing neuronal death in AMD are still controversial (Heesterbeek et al., 2020). There is growing evidence linking mitochondrial dysfunctions to AMD (Kaarniranta et al., 2020). With the progression of retinal degeneration, which may take several years or even decades, most AMD patients continually and irreversibly lose eyesight. The advanced AMDs include two subtypes: geographic atrophy (dry AMD) and choroidal neovascularization (wet AMD) (Heesterbeek et al., 2020). Anti-vascular endothelial growth factor (VEGF) therapy has been used to treat wet AMD, while there is no applicable therapeutic strategy for dry AMD (Solomon et al., 2019). Ideally, the most effective treatment for AMD is to prevent the disease aggravation in the early stage, which requires more investigations on initial alternations of both neuronal and non-neuronal cells in degenerating retinas.

The sophisticated roles of no-neuronal cells in retinal degeneration have been intensively investigated, especially for microglia, retinal pigment epithelium (RPE), and choroidal supporting cells (Zheng et al., 2010; Kumari et al., 2022; Voigt et al., 2022). As the most predominant glial subtype in the mammalian retina, Müller cells support the activities of photoreceptors and other retinal neurons by buffering potassium ions, removing neurotransmitters, secreting neurotrophic factors, and producing antioxidants (Bringmann et al., 2009a,b; Eastlake et al., 2020). These functions are high energy consuming, requiring substantial production of ATP. However, the role of mitochondrial functions in Müller cells is still under debate (Toft-Kehler et al., 2017). Although some early studies have claimed that glycolysis is the major contributor to energy production, recent research reported the considerable entry of labeled glycolytic carbon into the mitochondrial tricarboxylic acid cycle in Müller cells (Singh et al., 2020), suggesting the important contribution of ATP produced by OXPHOS. Up to now, the expression profiles of mitochondria-related genes encoded by mtDNA and nDNA in Müller cells are largely unknown. Additionally, Müller cells always go through morphological and/or physiological changes in response to retinal damage, leading to opposite functional consequences, either neuroprotective or neurotoxic (Bringmann et al., 2009a). Several studies have indicated the possibility to employ the “reactivated” Müller cells as a therapeutic target for AMD (Devoldere et al., 2019; Eastlake et al., 2020). However, current knowledge of the Müller cells’ role in AMD are largely from animal models, which may not be able to mimic the key phenotypes of human patients. More investigations in human samples are urgently needed.

In this study, we integrated recent published Single cell RNA-Seq (scRNA-Seq) datasets derived from human retinas with or without Early AMD to address the following questions. Whether individual Müller cells in Early AMD have different functional statuses of mitochondria and OXPHOS especially? Could the metabolic-mitochondrial state of a Müller cell determine its role in retina degeneration? And if so, what factors contribute to the key regulatory network of Müller cell reactivation?

## Materials and methods

### Human samples of retinal macula for analysis

This study involved 11 samples of human macula from three dataset series (GSE137537, GSE188280, and GSE203499) (Menon et al., 2019; Zauhar et al., 2022), including retinas from four AMD patients and seven healthy controls without degeneration. The details of each donor were listed in Table 1.

**TABLE 1:** Details of human retinal specimen.

Retina	Phenotype	Age	Gender	GEO accession
1	AMD	84	F	GSM5676874
2	AMD	94	M	GSM6173970
3	AMD	81	F	GSM6173978
4	AMD	99	F	GSM6173999
5	Ctrl	76	F	GSM6173966
6	Ctrl	84	M	GSM6173982
7	Ctrl	88	F	GSM6173986
8	Ctrl	86	F	GSM6173992
9	Ctrl	86	M	GSM4081526
10	Ctrl	71	M	GSM4081528
11	Ctrl	68	M	GSM4081524

### Data integration and processing

The raw sequenced reads were alimented to human genome (GRCh38) and counted by Cell Ranger (Version 7.0.1, 10xGENOMICS, Pleasanton, CA, USA). Then the count matrixes were input into R package Seurat (Version 4.1.1) and cells that have unique gene counts more than 2,000 or less than 300 were filtered. To keep more information for analysis of metabolic status, we took a loose criterion for percentage of mitochondrial genes (<10%). Multiple datasets were normalized and integrated by the functions “FindIntegrationAnchors” and “IntegrateData,” as previously described (Stuart et al., 2019). The data then were scaled and processed to principal component analysis for dimensionality reduction and clustering. The cell identity of each cluster was annotated by known markers. The Chi-square test for alternations of subcluster abundances was conducted by function “chisq.test” in R. R-studio (Version 1.4.1106) was employed to run all R packages and functions in this study.

### Calculation of proliferation probability

The proliferation probability (PP) of each cell was computed based on 51 critical cell-cycle genes reported in the previous study (Yang et al., 2021). The proportion of proliferative cells in each population, which was estimated by the percentage of Proliferating Cell Nuclear Antigen (PCNA) positive cells (Kisielewska et al., 2005), was employed as the prior probability for Bayes’ theorem. The original R-based codes provided by the authors of the original manuscript (Yang et al., 2021) were downloaded from the GitHub repository^1^ and directly used for the calculation of PP.

### Analysis of DEGs and GO-term enrichment

To access the differentially expressed genes among subclusters, the function “FindMarkers” in Seurat package was used. The adjusted *p*-value based on Bonferroni correction was employed to identify the statistical significance. Only the genes with more than twofold change were kept for further analysis. The Gene Ontology (GO)-term enrichment analyses for up/down regulated genes were performed by R package WebGestalt R (Version 0.4.4) (Liao et al., 2019). The “geneontology_Biological_Process_noRedundant” was used as the functional category for analysis. The significant threshold for False Discovery Rate (FDR) was 0.1.

### Multi-step upstream transcriptional regulator enrichment analysis

The possible transcription factors regulating the functional status of different subclusters of Müller cells were enriched by transcriptional regulator enrichment analysis (TREA) (Burda et al., 2022). Briefly, four resource databases, including ChIP enrichment analysis (ChEA), JASPAR CORE database, Transcription Factor database (TRANSFAC), and Ingenuity Pathway Analysis Upstream Regulator Analytic (IPA) were integrated to estimate the interaction between regulator–target genes. Then the Differentially Expressed Genes (DEGs) were used to predict the potential transcription factors that contribute to distinct expression profiles *via* direct DNA-binding or indirect regulation. The analysis was performed in Jupyter Notebook (Version 6.4.8) and the original codes were downloaded from https://github.com/burdalab/TREA.

## Results

### Single-cell transcriptomes reveal divergence of mtDNA expressions in Müller cells

**FIGURE 1:**
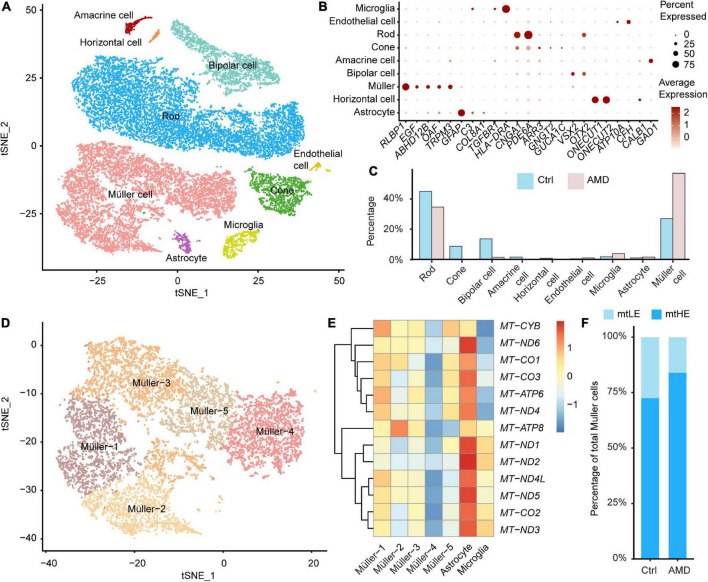
Single-cell transcriptomes of the human retina suggest the heterogeneity of Müller cells. **(A)** A total of nine neuronal and glial clusters in the human retina were identified and visualized by t-distributed Stochastic Neighbourhood Embedding (tSNE) dimensionality reduction. **(B)** The expressions of key marker genes help determine the cellular identification of each cluster. **(C)** The abundance of each cluster in samples from age-related macular degeneration (AMD) or healthy donors is different. **(D)** Müller cells are further divided into five subpopulations by unsupervised clustering. **(E)** The distinct glial cells residing in retinas have different expression profiles of mitochondrial encoded genes. The Müller-4 subcluster is marked as low expression of mtDNA (mtLE) cells. **(F)** The abundance of mtLE Müller cells is reduced in the AMD retina.

To investigate the disease-associated alternations of retina cells, we combined recently published databases of single-cell transcriptomes derived from maculas of AMD patients and healthy donors without retinal degeneration (total four cases of AMD and seven cases of control, see “Materials and methods”). After integration and data filtering, 22,597 cells were recovered and taken for the following clustering analysis (Figure 1A). The cell identities for each population were annotated according to known markers, including Müller cell (*RLBP1*, *EGF*, *ABHD12B*, *OAF*, and *TRPM3*), astrocyte (*GFAP*, *COL8A1*), microglia (*TGFBR1*, *HLA-DRA*), endothelial cell (*ATP10A*, *CFH*), rod photoreceptor (*CNGA1*, *PDE6A*), cone photoreceptor (*ARR3*, *GNGT2*, and *GUCA1C*), bipolar cell (*VSX2*, *OTX2*), horizontal cell (*ONECUT1*, *ONECUT2*), and amacrine cell (*GAD1*, *CALB1*) (Figures 1A, B). The retinal ganglion cells failed to be identified in the integrated database, which may be caused by the low cell abundance and cell loss during sample preparation.

In control samples, rod photoreceptors are the most abundant cells (45.0%), consistent with previous reports (Lukowski et al., 2019; Figure 1C). However, the percentages of retina glial cells, including Müller cells (27.0%), astrocytes (1.02%), and microglial cells (1.81%), are higher than expected (Figure 1C). The distorted glia-to-neuron ratio would be explained by that some neuronal cell types are more vulnerable to the enzyme-dependent dissociation process (Bakken et al., 2018). Also, it is possible that degenerative conditions in Early AMD retina further hinder the recovery of neurons for scRNA sequencing. Although overrepresented, the enriched glial cells facilitate our analysis of Müller cell-specific response to retina degeneration. Compared with healthy controls, the abundances of cell types in the degenerated macula are dramatically changed. The percentage of rod is lowered to 34.6%, and the cone portion is reduced from 8.72 to 0.21%, indicating the significant loss of photoreceptors in AMD. The proportions of other neuronal cells, including bipolar cells, horizontal cells, and amacrine cells are also decreased under the disease condition. In contrast, the abundances of glial cells are increased in AMD macula. Müller cells become the dominant cell population (57.0%) in AMD samples, demonstrating the reactive gliosis is associated with retina degeneration, as previously documented (Bringmann et al., 2009a). We thus focused on the disease-associated alternations of mitochondrial functions among Müller cells in the following study.

By further hypothesis-free clustering, total of 8,069 Müller cells were divided into five subpopulations, as shown in Figure 1D. We compare the expression of mtDNA between different subclusters of Müller cells and other retinal glial cells firstly (Figure 1E). Interestingly, a portion of Müller cells (Müller-4) show repressed expression of key mitochondrial coding genes (Figure 1E). Given none of these genes are involved in the unsupervised clustering analysis (see “Materials and methods”), our results indicate that the divergence of mtDNA expression may coordinate closely with the functional heterogeneity of Müller cells. Therefore, we designated Müller-4 as the low expression of mtDNA (mtLE) group, and all the other Müller cells were marked as mtHE (high expression of mtDNA). Next, we calculated the change of subpopulation abundance associated with AMD. As illustrated in Figure 1F, the proportion of mtLE Müller cells is reduced from 27.4 to 16.0% in AMD samples (Chi-square test, *p* < 2.2 × 10^–16^), suggesting the potential connection between retina degeneration and different subclusters of Müller cells with distinct mtDNA expression patterns.

### mtLE Müller cells are “sleep-like” quiescent

**FIGURE 2:**
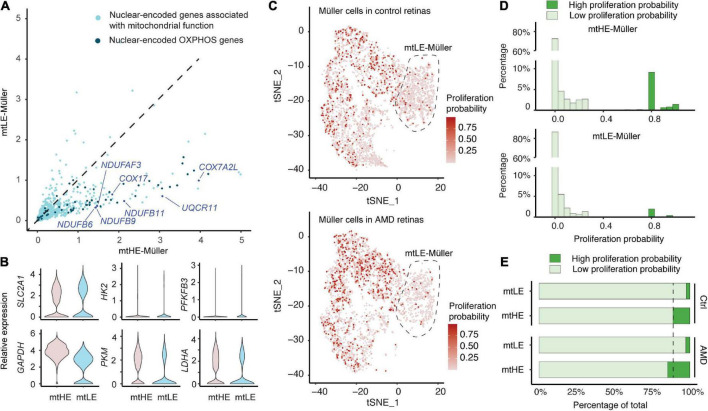
Low expression of mtDNA (mtLE) Müller cells exhibit low energy production and proliferative ability. **(A)** The expressions of mitochondrial genes encoded by nuclear DNA (nDNA) in mtLE Müller cells are repressed. The nuclear-encoded oxidative phosphorylation (OXPHOS) genes are highlighted by dark blue. **(B)** Key glycolysis genes are not upregulated in the mtLE subcluster. **(C)** The proliferation probability (PP) of each Müller cell is predicted based on expressions of key cell-cycle genes. **(D)** The histogram statistics of PP show that mtLE Müller cells are less proliferative. **(E)** The high expression of mtDNA (mtHE) Müller cells, but not the mtLE subpopulation, become more proliferative under age-related macular degeneration (AMD) conditions.

Because the assembly and activities of mitochondria are dependent on the protein products of both mtDNA and nuclear genome, we next ask whether the mtLE Müller cells also have reduced expressions of nDNA-encoded genes associated with mitochondrial functions. A total of 729 genes, containing 46 oxidative phosphorylation (OXPHOS) genes, were selected as previously documented (Mercer et al., 2011; Barshad et al., 2018). Compared with the mtHE population, expressions of numerous nDNA-encoded mitochondrial genes are suppressed in the mtLE Müller subcluster. Similarly, low expression levels of OXPHOS genes are also detected in these cells (Figure 2A). The consistently reduced expressions of mito/nuclear-encoded energy metabolism genes suggest the low function and energy production of mitochondrial respiration in mtLE Müller cells.

It has been reported that Müller cells merely rely on glycolytic ATP production (Toft-Kehler et al., 2017). Therefore, we quantified the expressions of six key glycolytic components in addition (Figure 2B), including glucose transporter 1 (*GLUL1*, encoded by *SLC2A1*), hexokinase 2 (*HK2*), 6-phosphofructo-2-kinase/fructose-2,6-bisphosphatase 3 (*PFKFB3*), glyceraldehyde 3-phosphate dehydrogenase (*GAPDH*), pyruvate kinase M1/2 (*PKM*), and lactate dehydrogenase A (*LDHA*). Interestingly, none of these genes is upregulated in the mtLE subcluster. Only *GLUL1* shows a slight upward trend (log_2_FC > 0.25) but fails to reach the statistical significance (*p*_adj_ = 1.0). In contrast, both *PKM* and *LDHA* are significantly reduced in the mtLE group (*PKM*: *p*_adj_ = 3.17 × 10^–135^;*LDHA*: *p*_adj_ = 1.73 × 10^–62^). Together, our results indicate that the mtLE group exhibits a suppressed metabolic state, which is different from other populations of Müller cells.

The cellular metabolic states always coordinate with regulation of cell cycle and proliferation (Intlekofer and Finley, 2019). Given the significant increase of Müller cell abundance in macula with AMD, we were wondering if the reduced energy production of mtLE subcluster also impacts the ability of proliferation upon retina degeneration. A recent published tool was employed to estimate the proliferation ability of each cell based on the expression of key cell-cycle genes revealed by scRNA-sequencing (Yang et al., 2021). In brief, a pool of 51 signature genes were selected and processed to calculate the proportion of cells in each phase. And by integration and estimation based on Bayes’ theorem, we were able to calculate the PP for each cell, ranging from 0 to 1. A higher score corresponds to a greater potential for cellular proliferation. The proliferation probabilities of each cell under different conditions are plotted in Figure 2C, showing the increase of proliferating Müller glia in AMD retinas. The frequency histograms demonstrate that most Müller cells are quiescent (PP < 0.05) both in mtLE and mtHE groups (Figure 2D), which is consistent with previous observations (Bringmann et al., 2009a; Hamon et al., 2019). However, more cells that have high PP (PP > 0.5) are detected in the mtHE subcluster, confirming the high energy demand of cellular proliferation. Additional quantifications show that the percentage of high PP cells is increased in mtHE groups, but not in mtLE subcluster (Figure 2E). Considering the upregulated mtHE subpopulation in AMD retina (see Figures 2C, E), it is reasonable to speculate that the expanded Müller glia population is more likely contributed by the mtHE cells with higher PP. Meanwhile, the mtLE subcluster represents a population of “sleep-like” quiescent Müller cells, characterized by suppressed energy production and low proliferation ability.

### The cellular metabolic states are coordinated with Müller cells’ functions

**FIGURE 3:**
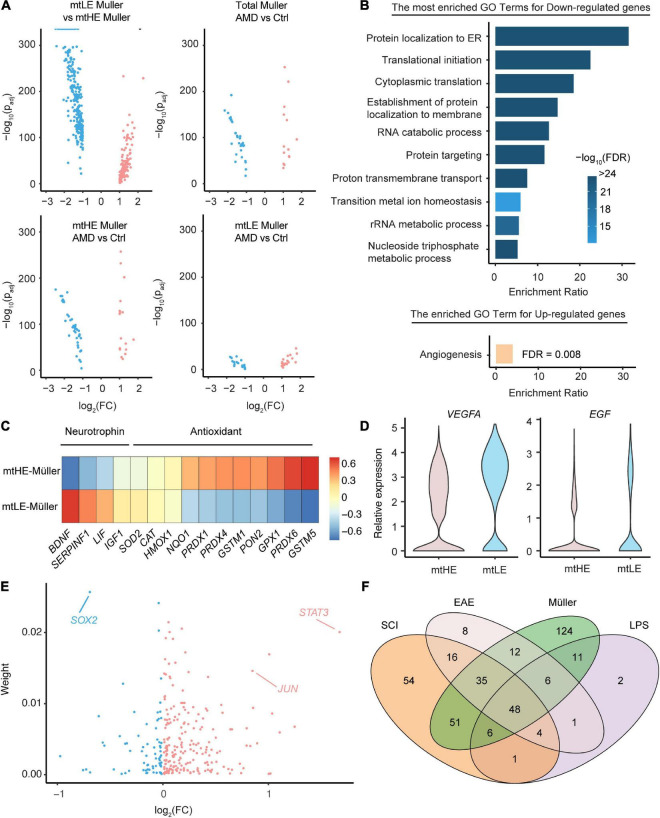
The functional diversity and regulatory networks of Müller cells with distinct metabolic-mitochondrial signatures. **(A)** The differential expression analysis enriched key DEGs under four comparison conditions. **(B)** The go-term analysis highlights the repressed protein synthesis in low expression of mtDNA (mtLE) Müller cells. Only the DEGs enriched by the comparison between mtLE and mtHE subpopulations [shown in the upper-left part of panel **(A)**] are processed in this analysis. **(C)** The mtLE Müller cells produce less antioxidant but more neurotrophin, compared with high expression of mtDNA (mtHE) subpopulation. **(D)** The expressions of *VEGFA* and *EGF* are upregulated in mtLE Müller cells. **(E)** The regulatory TFs controlling the expression profiles of mtLE and mtHE Müller cells are predicted. “Weight” indicates the estimated contribution of each TF and the *x*-axis is the fold change between mtLE and mtHE subpopulations. **(F)** The overlapping of regulators enriched from Müller cells and astrocytes under different conditions. SCI, spinal cord injury; EAE, experimental autoimmune encephalomyelitis; LPS, lipopolysaccharide.

Müller cells have versatile supportive functions in the healthy retina and play confounding roles during degeneration, which may orchestrate with mitochondria activities and metabolic states. To investigate whether the mtLE subcluster performs normal functions of Müller cells, we conducted the differential gene-expression analyses among distinct subpopulations. Interestingly, only the comparison between mtLE and mtHE subclusters enriches more than 400 DEGs (280 downregulated genes and 133 upregulated genes, see Supplementary Table 1), while the differences of expression profiles between Müller cells under control and AMD conditions are limited (total 47 DEGs) (Figure 3A). And the retina degeneration only caused 41 and 58 DEGs in mtLE and mtHE, respectively (Figure 3A). Therefore, our results suggest that the major change of Müller cells in AMD retinas is the altered abundances of distinct subpopulations, but there is no newly generated subcluster that can be termed as “reactivated glia.”

We next performed the Go-term enrichment analysis on the DEGs between mtLE and mtHE Müller cells (Figure 3B) to check their functional differences. The top GO terms enriched by downregulated genes in the mtLE subpopulation center on RNA translation and protein processing, including “protein localization to ER,” “translational initiation,” “cytoplasmic translation,” etc. Given protein biosynthesis is an energy-demanding process in cells, it is easy to understand the tied correlation between reduced protein production and suppressed mitochondria function in mtLE Müller cells. Interestingly, the only biological process enhanced in mtLE Müller cells is angiogenesis (Figure 3B). We further confirmed the functional divergence of different subclusters by plotting key genes related to the release of neurotrophins and antioxidants (Figure 3C), as well as angiogenesis (Figure 3D). Our results indicate that the levels of most antioxidants are reduced in mtLE Müller cells, while the expressions of certain neurotrophins are enhanced (Figure 3C). More importantly, we detected higher expressions of *VEGFA* (*p*_adj_ = 2.01 × 10^–115^) and *EGF* (*p*_adj_ = 2.01 × 10^–115^) in mtLE Müller cells, suggesting pro-angiogenic role of this subcluster (Figure 3D).

Finally, we wish to ask which transcription factors determine the distinct status of Müller cells with different metabolic-mitochondrial signatures. A newly published method, named multi-step upstream TREA, was engaged to address this issue. TREA has been successfully used to speculate the regulatory network for reactivation of astrocytes under different pathological conditions (Burda et al., 2022). By incorporating of public datasets and DEGs enriched in our study, the contributions of Transcription Factor (TF) candidates are estimated (Figure 3E and also listed in Supplementary Table 2). Strikingly, several TFs known as key regulators of glial response to injury (*STAT3*, *SOX2*, *JUN*, etc.) are also enriched in Müller cells. The expression differences of these factors between two subclusters are also significant (*STAT3*: *p*_adj_ = 1.07 × 10^–109^; *SOX2*: *p*_adj_ = 1.66 × 10^–95^; *JUN*: *p*_adj_ = 7.38 × 10^–63^). Therefore, we analyzed the overlap of Müller cell-enriched TFs and regulators involved in gliosis induced by lipopolysaccharide (LPS), experimental autoimmune encephalomyelitis (EAE), or spinal cord injury (SCI) (Figure 3F). The Venn diagrams show that numerous TFs regulating statuses of Müller cells (57.7%) also participate the astrocytic reactivation (Figure 3F), indicating the shared mechanisms determining glial responses to injury and neuronal degenerations in multiple regions of the central nervous system.

## Discussion

In this study, we reveal the diversified expression profiles of mitochondrial genes of Müller cells by analyzing scRNA-seq data of maculas from patients with AMD and healthy donors. Compared with a recent report (Huang et al., 2022), we conducted the cluster analysis from a novel perspective. A subcluster (mtLE Müller cell) with suppressed energy production is identified by low PP and reduced biosynthesis of proteins. Interestingly, the mtLE Müller cells exhibit enhanced expression of several growth factors, especially *VEGFA* and *EGF*, indicating their confounding roles in healthy and degenerating retina. Furthermore, by estimating the abundances and proliferation capability of different subpopulations, we speculate the roadmap of Müller cell reactivation in AMD retina, indicating the importance to understand the relationship between glial heterogeneous features and distinct cellular metabolic-mitochondrial signatures.

Although the vision loss of AMD is mainly caused by the degeneration of photoreceptors, the critical roles of supportive cells in neural death have been documented. For instance, it has been well demonstrated that oxidative stress leads to neuronal damage mediated by dysfunctions of RPE cells (Ferrington et al., 2020). Despite their various key functions, Müller cells were less noticed in AMD pathogenesis in previous studies (Ferrington et al., 2020). However, there is now plenty of evidence indicating that Müller cell are deeply involved in retinal degeneration by releasing of chemokine (Natoli et al., 2017), recruiting microglia (Rutar et al., 2012), and regulating retinal ion transport (Baumann et al., 2017), etc. Our study further shows that even in the early phase of AMD, Müller cells already exhibit measurable alternations.

The reactivation of glial cells in response to retinal injury has been identified both in animal models and patients. However, the functional consequences introduced by reactivated Müller glia are still controversial, which mainly depends on the causes and stages of retinal damage. In the early phase of gliosis, the proliferation of Müller cells may protect retinal neurons from an excitotoxic environment by enhanced potassium buffering, glutamate reuptake, and neurotrophin release (Toft-Kehler et al., 2017; Eastlake et al., 2020). In contrast, long-lasting and aggressive reactivation is always associated with the repressed normal function of Müller cells and the stimulated formation of glial scars. Therefore, one of the keys to developing Müller glia-based therapeutic for retinal degeneration is demonstrating the functional characteristics of gliosis tied to different stages of disease (Bringmann et al., 2009a). Interestingly, we failed to detect either a new “reactivated” subpopulation or the enhanced expression of *GFAP* among Müller cells in Early AMD retina, in contrast to the previous observation in autoimmune retinopathy (Voigt et al., 2020). Instead of “classical” reactivation, the major change of Müller glia is the alternation of subpopulation abundances: more mtHE cells are detected in degenerating retinas. Given that the neuroprotective functions of Müller cells are always energy-consuming, the increase of the mtHE subcluster may be a part of the anti-damage mechanisms in retinas, confirmed by the enhanced production of antioxidants in these cells. We failed to detect significant changes of genes encoded proteins for other critical functions of Müller cells, including ions and neurotransmitters removal, maybe due to the early stage of recruited samples.

Despite the general repression of protein synthesis, the mtLE Müller cells have higher expression levels of several growth factors, especially *VEGFA* and *EGF*. VEGF plays a key role in the abnormal angiogenesis in retina and the anti-VEGF medicine shows convincing therapeutic effects in patients with wet AMD. However, whether the Müller cell-derived VEGF also contributes to the pathogenesis of Early AMD is still unknown. The increased abundance of mtHE subcluster, which has lower expression of VEGFA, may slow down the disease progress toward wet AMD. Meanwhile, it may also impact the viability of multiple retinal neurons, considering the protective role of VEGF reported in previous researches (Fu et al., 2015; Froger et al., 2020). Due to the limitation of samples recruited in this study, we have no conclusion whether these cases in early stage will develop to wet AMD or not. The impact of mtLE Müller cells in AMD may be addressed by future studies including more samples representing different disease stages. Another potential limitation of our study is the modest difference of ages between AMD and healthy donors. As recently reported (Huang et al., 2022), both the mitochondrial functions and VEGF expressions are alternated in old human retina. The effects of aging on Müller cells may need further investigation.

In the final part of our analysis, we employed a recently published bioinformatic tool to predict the regulatory network determining the function statuses associated with different metabolic-mitochondrial signatures in Müller cells. Interestingly, numerous transcription factors controlling the typical astrocytic reactivation are also enriched in Müller cells. The most notable one is *STAT3*, which is upregulated in mtLE subpopulation. Together with NF-κB pathway, STAT3 is reported as a key regulator controlling the glial response to injury in Central Nervous System (CNS) and shaping different subpopulations of reactivated astrocytes (Liddelow et al., 2017; Clarke et al., 2018). For example, STAT3 may direct astrocytes to form glial scar upon acute trauma and promote axonal regeneration (Anderson et al., 2016). Regarding to the functional similarities between these two astroglial subtypes (Blank et al., 2021), it is not surprising that STAT3 may also play a critical role in Müller cells. Although the exact regulatory networks controlled by STAT3 in retinal glial cells are yet largely unknown, we are still able to find evidence suggesting the potential relationship between this key TF and functional features of mtLE subpopulation from previous studies conducted in other types of cells. For instance, it has been documented that STAT3 may suppress the mitochondrial activity in fibroblast and epithelial cancer cell lines (Demaria et al., 2010), coinciding with the low expression levels of OXPHOS genes in mtLE Müller cells. Furthermore, the upregulation of VEGF mediated by the activation of JAK2/STAT3 signaling pathway has been demonstrated in non-small-cell lung cancer (Zhao et al., 2011) and other tumor cells, indicating that a similar correlation may also exist in mtLE subpopulation. However, whether the inhibition of proliferation of mtLE Müller cells is determined by STAT3 or other key regulators requires more investigation, given most articles have reported the pro-proliferative effect of STAT3.

In conclusion, we demonstrate the functional heterogeneity of Müller cells in healthy and degenerating human retina by integrating multiple datasets of scRNA-seq. The statuses of mitochondria and OXPHOS especially of these glial cells are closely correlated with their functions and roles in Early AMD. Our study suggests the potential association between distribution of Müller glial subcluster and early phase of retina degeneration, highlighting the significance of further investigating mitochondrial-based therapeutic interventions for AMD.

## Data availability statement

The original contributions presented in this study are included in the article/Supplementary material, further inquiries can be directed to the corresponding authors.

## Ethics statement

The studies involving human participants were reviewed and approved by the Human Research Ethics Committee of the Sichuan Academy of Medical Sciences and Sichuan Provincial People’s Hospital. Written informed consent for participation was not required for this study in accordance with the national legislation and the institutional requirements.

## Author contributions

All authors listed have made a substantial, direct, and intellectual contribution to the work, and approved it for publication.

## Footnotes

1
https://github.com/tianchengzhe/drug_response_scripts
